# Cross-talk of inflammation and cellular senescence: a new insight into the occurrence and progression of osteoarthritis

**DOI:** 10.1038/s41413-024-00375-z

**Published:** 2024-12-03

**Authors:** Zeyu Han, Ketao Wang, Shenglong Ding, Mingzhu Zhang

**Affiliations:** Department of Foot and Ankle Surgery, Beijing Tongren Hospital, Capital Medical University, 100730 Beijing, PR China

**Keywords:** Pathogenesis, Diseases

## Abstract

Osteoarthritis (OA) poses a significant challenge in orthopedics. Inflammatory pathways are regarded as central mechanisms in the onset and progression of OA. Growing evidence suggests that senescence acts as a mediator in inflammation-induced OA. Given the lack of effective treatments for OA, there is an urgent need for a clearer understanding of its pathogenesis. In this review, we systematically summarize the cross-talk between cellular senescence and inflammation in OA. We begin by focusing on the mechanisms and hallmarks of cellular senescence, summarizing evidence that supports the relationship between cellular senescence and inflammation. We then discuss the mechanisms of interaction between cellular senescence and inflammation, including senescence-associated secretory phenotypes (SASP) and the effects of pro- and anti-inflammatory interventions on cellular senescence. Additionally, we focus on various types of cellular senescence in OA, including senescence in cartilage, subchondral bone, synovium, infrapatellar fat pad, stem cells, and immune cells, elucidating their mechanisms and impacts on OA. Finally, we highlight the potential of therapies targeting senescent cells in OA as a strategy for promoting cartilage regeneration.

## Introduction

The aging population presents a significant and imminent challenge to global society. By 2050, individuals over 60 years old are projected to make up approximately 22% of the global population.^1^ The aging body becomes increasingly susceptible to age-related chronic diseases, including chronic heart failure, atherosclerosis, chronic obstructive pulmonary disease, diabetes, and osteoarthritis (OA).^2–4^ Due to the gradual loss of internal homeostasis in aging organisms, effective defenses fail to activate in response to internal and external stressors. Senescence, an intricate and interconnected process, manifests universally in biological organisms, regardless of a clear correlation between biological and chronological aging.^5^

Senescence is marked by a progressive loss of function at the tissue and cellular levels. Aging is driven by the accumulation of senescent cells at the cellular level. First introduced by Leonard Hayflick and Paul Moorhead in 1961, the concept of “senescence” suggests a correlation between cellular senescence and biological aging.^6^ Notably, senescence can be classified into three types in vivo: acute, embryonic, and chronic senescence.^7,8^ Unlike the former two, cells entering senescence due to chronic stimuli exert harmful effects on the organism. This review focuses on this form of senescence. Inflammation, the most common source of chronic stimulation, follows an age-related pattern, with longitudinal studies revealing a tendency for chronic low-grade inflammation with age.^9^ Comparing inflammation-related markers in the lungs of mice at different ages shows a pro-inflammatory shift in the lungs of aged mice.^10^ A unique inflammatory cell profile in adipose tissue has been identified in aged mice.^11^ These findings indirectly suggest an link between inflammation and senescent cells, with studies attempting to uncover their causal relationship.^12,13^ Consequently, the term “inflammsenescence” was coined to describe the heightened pro-inflammatory state during senescence.^14^

**Fig. 1:**
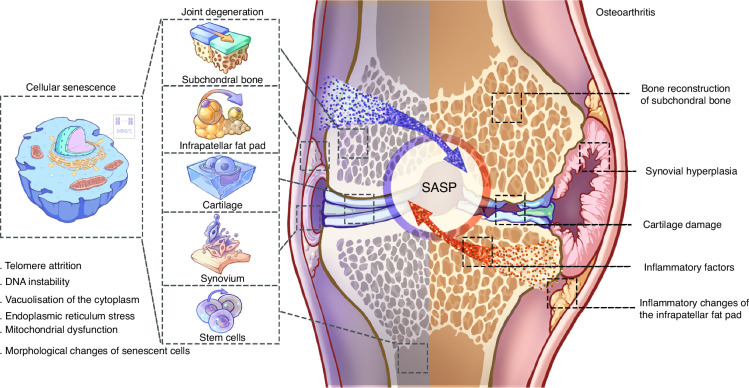
Schematic diagram of the cross-talk between cellular senescence and OA. Characteristic manifestations of joint degeneration are as follows: senescent subchondral bone with decreased bone density, reduced bone mass and thinning, senescent infrapatellar fat pad with inflammatory changes, senescent chondrocytes with decreased function accompanied by increased matrix degradation, senescent synoviocytes with diminished function and chronic synovitis, and senescent stem cells with diminished function and genetic stability. Alterations in senescent cells include telomere attrition, DNA instability, vacuolization of the cytoplasm, endoplasmic reticulum stress, mitochondrial dysfunction and alterations in the cell membrane. The characteristic pathology of OA includes reconstruction of the subchondral bone, synovial hyperplasia, cartilage damage, and inflammatory changes in the infrapatellar fat pad. The inflammatory mediators form a bridge of communication between cellular senescence and OA, and senescent cell-associated secretory phenotypes (SASP) play a critical role in this process

Osteoarthritis (OA), a joint disorder characterized by chronic pain and cartilage deterioration, manifests as a persistent low-grade inflammatory condition.^15,16^ Current clinical treatments for OA include oral nonsteroidal anti-inflammatory drugs, joint injections of hyaluronic acid, and surgical interventions. However, none can halt the pathological process.^17^ With an aging population, early personalized prevention is emerging as a key intervention, focusing on avoiding risk factors such as obesity, joint damage, and impaired muscle function.^18^ However, senescence, an additional risk factor for OA, is difficult to avoid and plays a significant role in the disease process.^19^ In this review, we summarize the cross-talk between cellular senescence and inflammation in OA (Fig. 1). First, we focus on the mechanisms and hallmarks of cellular senescence, summarizing evidence supporting the correlation between cellular senescence and inflammation. We then discuss the mechanisms underlying the interaction between cellular senescence and inflammation. Additionally, we examine various types of cellular senescence in OA, including senescence in cartilage, subchondral bone, synovium, infrapatellar fat pads, and stem cells. Furthermore, we summarize the senescence of stem and immune cells, elucidating their mechanisms and impacts within OA. Finally, we highlight the potential of targeting senescent cells as a therapeutic approach for OA, offering new strategies to promote cartilage regeneration.

## Aging and cellular senescence

Organisms experience an irreversible aging process marked by a gradual loss of physiological integrity, reducing their ability to withstand internal and external stressors and increasing their susceptibility to injury and disease.^20^ Senescence reflects the culmination of changes over an organism’s lifetime, evidenced by the accumulation of senescent cells in aging tissues.^21,22^ Therefore, elucidating cellular senescence is crucial for understanding the processes associated with aging and its pathology.

Cellular senescence was first observed around 60 years ago when Leonard Hayflick and Paul Moorhead discovered that human fibroblasts degenerate after approximately 50 passages and a year in culture, a phenomenon termed the “Hayflick limit,” which represents replicative senescence.^6^ Premature cellular senescence, which occurs before telomere shortening, has also gained attention and includes stress-induced senescence, oncogene-induced senescence in vitro, and tumor suppressor deficiency-induced senescence.^23^ Regardless of the trigger for cellular senescence, the ultimate outcome is cell cycle arrest.

### Hallmarks of aging

Numerous studies have extensively documented the hallmarks of aging, despite some variations. These hallmarks share common characteristics, including time dependence, aging-promoting effects, and potential therapeutic applications.^24,25^ López-Otín et al. systematically summarized nine hallmarks of aging as early as 2013: DNA instability, telomere attrition, epigenetic alterations, loss of proteostasis, dysregulated nutrient sensing, mitochondrial dysfunction, cellular senescence, stem cell exhaustion, and altered intercellular communication.^20^ Subsequently, two additional hallmarks, chronic inflammation (inflammaging) and ecological dysregulation, were added to highlight the critical role of inflammation in aging, which is the primary focus of this review.^26^

### Characterization of senescent cells

**Fig. 2:**
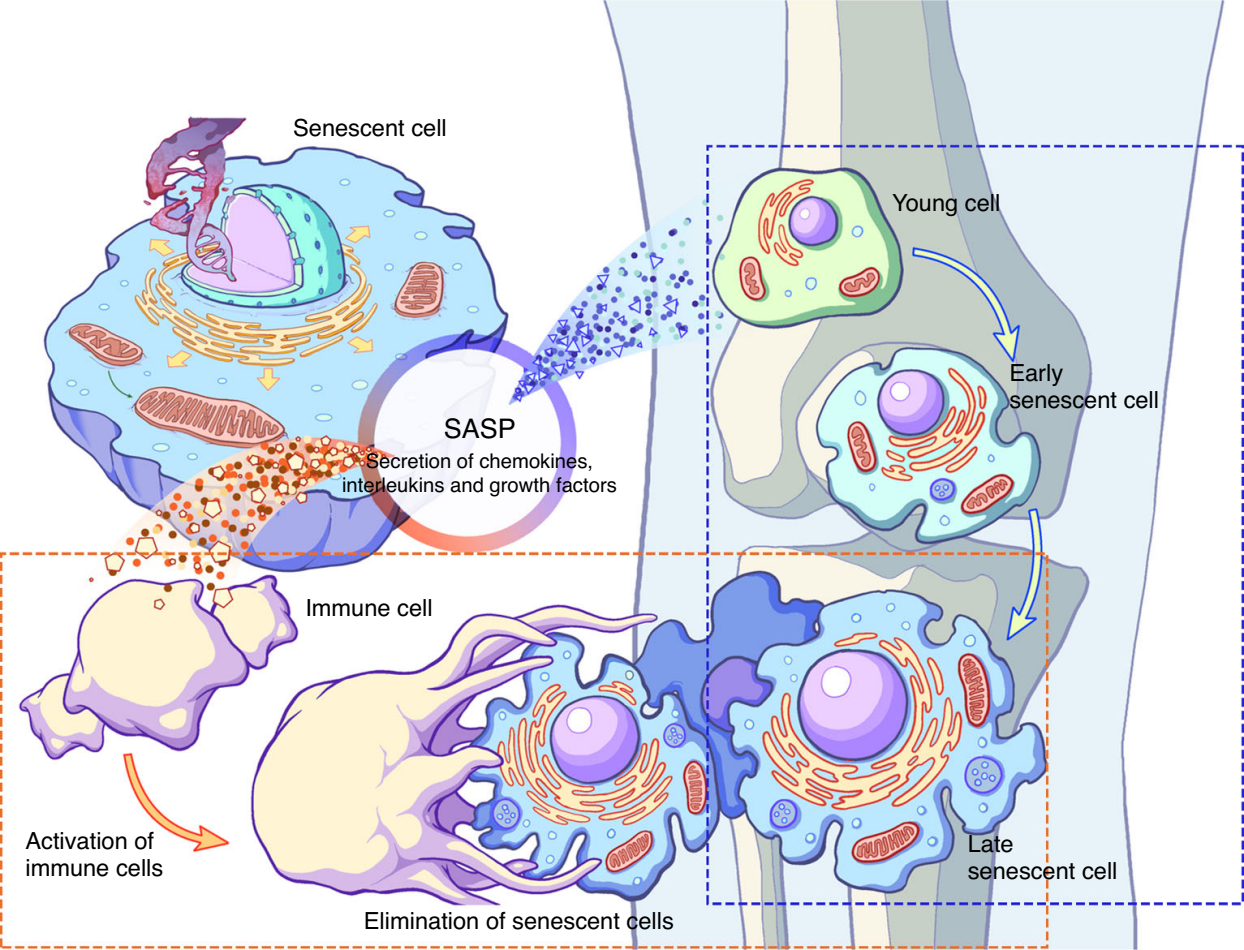
Schematic overview of the role of SASP in senescent cells. Senescent cells secrete interleukins and chemokines, which act on neighboring cells in a paracrine manner. SASP factors act on neighboring cells to induce a late senescent state among young and early senescent cells promoting a senescent microenvironment. In contrast, by activating immune cells to eliminate senescent cells, SASP establishes a connection with the immune system to participate in tissue repair and remodeling

Senescent cells are characterized by altered cell morphology, telomere shortening, and a range of distinctive senescence markers, as shown in Table 1. Another key feature is the shift in secretory phenotype observed in senescent cells, first identified by Krtolica et al.^27^ through the detection of soluble and insoluble factors secreted by senescent human fibroblasts. This phenomenon, known as the senescence-associated secretory phenotype (SASP), is marked by increased pro-inflammatory mediators (IL-1, IL-6, IL-7, IL-8, IL-18, TNF-α) and matrix metalloproteinases (MMP1, MMP10), significantly influencing the microenvironment (Fig. 2).^28–31^ Notably, although SASP maintains senescent cell cycle arrest, its regulation does not appear to be associated with cell cycle arrest.^32,33^ Unexpectedly, SASP plays a positive role in linking with the immune system by activating immune cells via paracrine effects to clear senescent cells (Fig. 2).^34,35^ In conclusion, senescent cells secrete a large number of bioactive molecules via SASP, which significantly impact their microenvironment and play key roles in various pathological processes, including tissue aging, chronic diseases, and cancer.

**Table 1:** Common cellular senescence markers

Marker	Tendencies	Specificities	Ref.
Senescence-associated-β-galactosidase (SA-β-Gal)	↑	The most frequently employed senescence marker and relevant to the lysosomal stress response	^25,29^
Senescence-associated heterochromatic foci (SAHF)	↑	Not suitable for detecting aging alone, needs to be combined with other markers	^214^
SASP	↑	Most senescent cells secrete related factors	^42^
H2A.J	↑	Only found in mammals, accumulates in fibroblasts	^215^
Lamin B1	↓	The functional relationship is not yet clear, and it varies with research	^114,216^
High mobility group box 1	↓	may be stress-induced, manifests as nuclear exclusion	^217^
MicroRNA-146a/9/204/367	↑	Associated with vascular remodeling, predicts inflammation-related genes	^218,219^
Cell cycle regulators			
p16^INK4a^	↑	Antiproliferative bioactivity	^220,221^
p53/p21^CIP1^	–	Not necessarily expressed, depending on the type of aging program	^222^
DEC1/DEC2	↑	Co-localization with the senescence marker SA-β-Gal	^223^
MSC-derived microvesicles (MSC-MVs)	–	Senescent late passaged MSCs secrete higher levels and smaller sized MSC-MVs	^224^

### Mechanism of SASP

The SASP in senescent cells is generated and maintained by complex regulatory mechanisms. Key signaling pathways, such as NF-κB, JAK2/3, and p38 MAPK, play pivotal roles in initiating SASP.^7,36^ Sustained DNA damage, along with GATA4 suppression, has been identified as an inducer of SASP.^9,37^ Cytoplasmic chromatin fragments in senescent cells trigger SASP by activating the cytoplasmic DNA-sensing cGMP-AMP synthase-STING (cGAS-STING) pathway of innate immunity. The presence of topoisomerase 1-DNA covalent cleavage complexes in cytoplasmic chromatin is essential for this process, suggesting the role of conserved features of innate immunity in senescence.^38–41^ Victorelli et al. revealed a mechanism by which mitochondria regulate SASP: in senescent cells, a small fraction of mitochondrial outer membranes is permeabilized, requiring BAX and BAK macropores to release mitochondrial DNA into the cytoplasm.^42^ Cytoplasmic mitochondrial DNA (mtDNA) then activates the cGAS-STING pathway. Yasuda et al. found that pro-inflammatory cytokine-driven downregulation of EZH2 maintains SASP via demethylation of the H3K27me3 marker in cancer-associated fibroblasts.^43^ Non-classical monocytes accumulate in aged individuals with elevated plasma TNF-α and IL-8 levels. The highly pro-inflammatory nature of non-classical monocytes may be a manifestation of SASP, induced by elevated levels of phosphorylated NF-κB (p65).^44^ Senescent cells maintain and regulate SASP through these mechanisms, which play a key role in shaping the tissue microenvironment.

## Cross-talk between inflammation and cellular senescence

The impact of cellular senescence on inflammation has been previously discussed. Here, we focus on the reciprocal effects of inflammation on cellular senescence. Inflammation precedes senescence and is a better predictor of senescence onset than telomere length.^45^ Senescent cells secrete pro-inflammatory mediators, which in turn influence cellular senescence. This phenomenon, occurring when inflammation is excessively regulated, is known as inflammsenescence.^13,46,47^ Senescent chondrocytes are consistently observed in OA cartilage.^29^ Although low-grade inflammation may exist in normal joints, the presence of senescent chondrocytes in OA cartilage strongly suggests that inflammation plays a pivotal role in driving cellular senescence. Similarly, anti-inflammatory phenotypes have been found in centenarian cells, where longevity-associated activation of transcription factor 7 is upregulated, indicating that inflammation plays an important role in cellular senescence.^48,49^ These findings suggest a correlation between inflammatory mediator levels and the expression of senescence markers.^50^ Accordingly, we discuss the effects of inflammation on cellular senescence, focusing on the opposing aspects of inflammation and anti-inflammation.

### Inflammation promotes cellular senescence

**Fig. 3:**
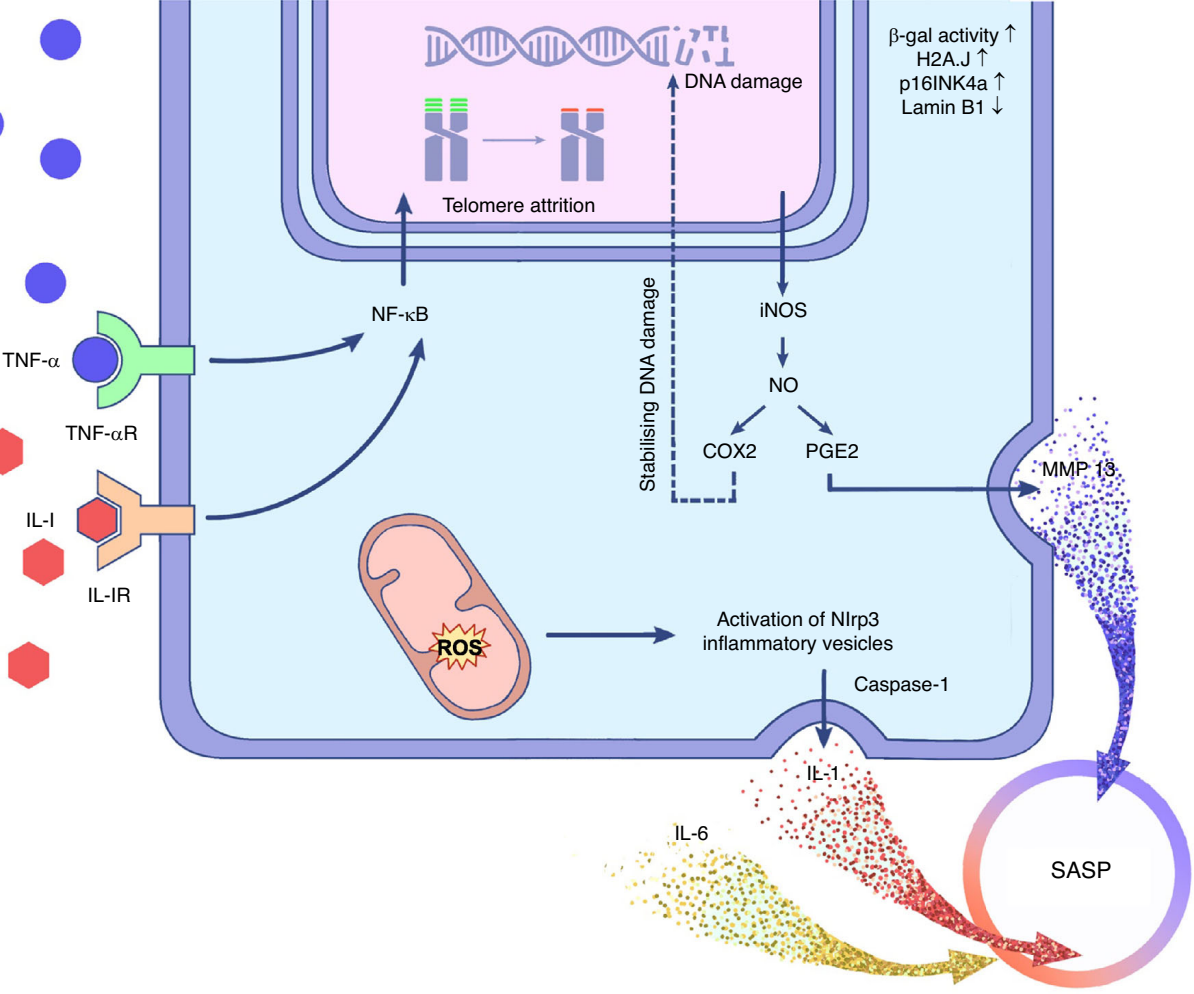
Schematic illustration of the mechanisms by which inflammation regulates cellular senescence. DNA damage and telomere attrition are associated with cellular senescence, accompanied by elevated senescence markers. Prolonged exposure of cells to a microenvironment comprising inflammatory factors such as IL 6 or IL 8 transduces signals into the cell interior via receptors on the cell membrane. Increased ROS activate the Nlrp3 inflammatory vesicle, regulate the activation of caspase-1 molecules upstream of inflammatory mediators, and increase the synthesis of SASP factors in senescent cells. Inducible nitric oxide synthase (iNOS) produces large amounts of nitric oxide (NO), increases prostaglandin E2 (PGE2) and cyclooxygenase (COX2) synthesis, further increases matrix metalloproteinase synthesis, and stabilizes DNA damage

Inflammation promotes senescence through pathways such as immune system overstimulation, leading to immunosenescence, tissue degradation, and disruption of stem cell function.^51^ However, the molecular mechanisms underlying this process remain unclear (Fig. 3). Ribeiro et al. found that, even without a pro-inflammatory lipopolysaccharide attack, indoxyl sulfate alone induced low-grade inflammation via macrophages, while promoting senescence in renal tubular epithelial cells during injury.^52^ Overexpression of eotaxin-1/CCL11 increases senescence markers such as CDKN2A (p16INK4a) and SERPINE1 in airway epithelial pneumocytes via pro-oxidative and pro-inflammatory pathways.^53^ Strong evidence clarifying the molecular mechanisms of how inflammation promotes cellular senescence is lacking, and most studies focus on elucidating this mechanism based on inflammatory factor levels. Impaired endothelium produces IL-1β, driving inflammation in the stromal niche and leading to hematopoietic senescence characterized by skewed stem cell differentiation, which can be ameliorated by blocking IL-1β.^54^ Exposure to pro-inflammatory cytokines IL-6 and IL-8 induces a self-perpetuating senescent microenvironment, increasing breast cancer cell invasiveness.^55^ An investigation demonstrated that prolonged exposure of MCF cells to IL-6 or IL-8 induced senescence, a process that could be reversed using a neutralizing antibody.^56^ However, higher concentrations of IL-6 and IL-8 failed to induce cellular senescence; only when cells were moderately damaged or in a near-senescent state could inflammatory factors promote senescence.^57^ These observations highlight the complexity of the relationship between inflammatory factors and the induction of cellular senescence, demonstrating the multifaceted and tightly regulated nature of this interaction.

Pro-inflammatory factors (e.g., IL-1β and TNF-α) do not act alone in promoting senescence; they stimulate the accumulation of reactive oxygen species (ROS), which synergistically accelerates the deterioration process.^43^ Notably, Yagi et al. found that ROS play a crucial role in inflammation-induced cellular senescence.^58^ Cells with telomeric wear in the plasma of young leukemia patients exhibit senescent biology, associated with elevated inflammatory cytokines and ROS-induced telomeric DNA damage.^5,59^ Knockout of the nfkb1 subunit induces chronic, low-level inflammation, leading to premature aging in mice. The underlying mechanism involves DNA damage, stabilized by increased NF-κB, COX-2, and ROS.^60^ Although consensus on the precise mechanisms of how inflammation promotes cellular senescence remains elusive, it is widely acknowledged that ROS-mediated DNA damage plays a role.

As a major source of inflammatory mediators in vivo, lipids accelerate inflammation, thereby promoting senescence.^61–63^ Chronic auto-inflammation triggered by adipocytes in RECC1-deficient mice plays a key role in adipose tissue degeneration, leading to premature senescence.^64^ A negative correlation between plasma lipid levels and telomere length was found in patients with Cushing’s syndrome, with a more pronounced effect in obese patients.^65^ The strong correlation between lipids and immune senescence is attributed to adipose tissue producing a subpopulation of pro-inflammatory B-cells, which induces the expansion of pro-inflammatory T-cells, accelerating immune senescence.^66,67^ The inflammatory phenotype of adipose tissue is linked to macrophages. Therefore, limiting macrophage numbers and their antigen presentation function can reduce adipose tissue inflammation to some degree.^68–70^

### Anti-inflammatory strategies inhibit cellular senescence

Medication is one of the most common strategies for inhibiting cellular senescence. Cellular senescence can be suppressed by anti-inflammatory drugs. Nonsteroidal anti-inflammatory drugs (NSAIDs) have been shown to rescue telomere dysfunction in mice with premature senescence induced by knockout of the nfkb1 subunit.^60^ Similarly, the lifespan of genetically heterogeneous wild-type mice was extended by long-term aspirin use.^71^ Although 17α-estradiol has minimal effects on senescent cells, it significantly extends lifespan in mice by reducing adipose tissue inflammation.^72^ Schroer et al. recently found that platelet factor 4 (PF4) levels in mouse and human plasma negatively correlate with age.^73^ They also showed that treating aged male mice with plasma from young mice significantly reduced hippocampal neuroinflammation, ultimately rescuing cognition. Increased hyaluronan levels were observed in several tissues of transgenic mice overexpressing the naked mole rat hyaluronan synthase 2 gene, along with a significant reduction in tissue inflammation. This led to prolonged lifespan and improved health, attributed to the anti-inflammatory properties of macromolecular hyaluronan.^74^ These conclusions suggest that inflammation levels can be regulated by anti-inflammatory drugs, thereby inhibiting cellular aging. In the later sections of this review, we will summarize the use of medications, including anti-inflammatory drugs.

Exercise or diet can also inhibit cellular senescence by reducing inflammation. Exercise training significantly suppressed inflammatory signaling in the hippocampus and increased Fas- and mitochondria-dependent apoptosis.^75^ Interestingly, older rats showed elevated levels of inflammatory proteins after swimming exercise alone, which the authors attributed to the intensity of the exercise. A 5-year follow-up showed that anti-inflammatory diets reduced mortality from aging-associated chronic diseases, a mechanism linked to the maintenance of telomere length.^76^ Furthermore, a large population-based cohort study found an association between pro-inflammatory diets and low-grade inflammation, increasing the risk of chronic diseases.^77^ These findings suggest that anti-inflammatory approaches may delay or even reverse cellular senescence, offering new avenues for future research aimed at mitigating cellular senescence.

## Inflammatory respones in tissue healing and repair

The body’s defense mechanisms initiate inflammation as an adaptive response to harmful stimuli, such as infections and tissue damage.^78^ The inflammatory response is a tightly regulated and precise process. Upon exposure to a harmful stimulus, the first step is detecting the stimulus via cell surface pattern receptors. These receptors include pathogen-associated molecular patterns (PAMPs) that activate germline-encoded pattern-recognition receptors (PRRs) in immune and nonimmune cells. Danger-associated molecular patterns (DAMPs) are also recognized by PRRs in response to signals released during tissue or cellular injury.^79^ Several intracellular signaling pathways, including nuclear factor kappa-B (NF-κB) and mitogen-activated protein kinase (MAPK), are activated upon receptor activation.^80,81^ Activation of inflammatory cells, like macrophages and adipocytes, triggers the release of inflammatory markers, including cytokines (e.g., interleukins, colony-stimulating factors, IFNs, TNFs, TGFs, and chemokines). Additionally, the coordinated network of multiple cell types recruits activated macrophages, monocytes, and other cells to the site of tissue injury or infection.^82^

Tissue damage from traumatic injury often leads to cell death. Unlike apoptosis, necrosis is more likely to cause cell membrane disruption.^83^ Inflammatory stimuli include various molecules released from necrotic cells, such as DNA, RNA, histones, and heat shock proteins, collectively known as DAMPs. Additionally, damaged cells release cytokines like interleukin 1a (IL-1a) and interleukin-33 (IL-33), known as alarmins. Alarmins induce immune cell migration, while DAMPs induce immune cell activation. Precise coordination between inflammatory and tissue-specific cells is crucial for restoring injured tissue and maintaining homeostasis in vivo. While the regulatory mechanisms behind this process remain unclear, a well-regulated inflammatory response is essential for tissue repair. The type 2 immune response plays a key role in limiting the reparative component of acute tissue injury. Additionally, a regulated inflammatory response prevents fibrosis. However, if the inflammatory response triggered by tissue injury is uncontrolled, it can lead to fibrosis and impaired function, especially in chronic inflammation.

## The molecular and cellular mechanisms of inflammation in OA

### Inflammatory responses in OA

Although OA is classified as an aseptic “non-inflammatory” arthropathy, its inflammatory response is complex, extending beyond the cartilage to the subchondral bone, synovial membrane, and infrapatellar fat pads.^84,85^ In addition to activated macrophages and neutrophils, chondrocytes and fibroblast-like synoviocytes play important roles in the process.^86,87^ Joint-resident cells, along with immune cells stimulated by DAMP, co-regulate the inflammatory network.^88^

Cytokines and chemokines, including pro-inflammatory cytokines IL-6, IL-8, IL-15, and IL-33, are secreted by the above cells. The secretion of these pro-inflammatory cytokines increases with DAMP expression. Inflammation-triggering mediators IL-1β and TNF-α are secreted in the early stages of OA.^89^ TNF-α stimulates TNF receptor 1 (TNFR1) and TNF receptor 2 (TNFR2), activating downstream signaling pathways. Notably, both receptors are expressed in synovial membranes, with TNFR1 strongly inducing proinflammation and TNFR2 capable of eliciting both proinflammatory and anti-inflammatory effects depending on the pathology.^90–92^ These pro-inflammatory factors stimulate the production of large amounts of nitric oxide (NO) by inducible nitric oxide synthase (iNOS), which in turn increases prostaglandin E2 (PGE2) and cyclooxygenase (COX2) synthesis. Meanwhile, PGE2 increases MMP13 production, leading to collagen degradation (Fig. 3).^93–95^ Pro-inflammatory cytokines activate the inflammatory response in surrounding cells, further sustaining SASP. The prolonged presence of SASP, in turn, exacerbates chronic inflammatory responses, creating a feedback loop.

Anti-inflammatory cytokines, including IL-4, IL-10, and IL-37, act as negative regulators, with IL-37 inhibiting M1 polarization and IL-33 promoting it. Rai et al. in their analysis of knee and hip cartilage from OA patients, found that increased IL-37 expression inhibited macrophage conversion to the M1 phenotype, while IL-33 had the opposite effect.^88^ These findings suggest that interactions between pro-inflammatory and anti-inflammatory cytokines, along with macrophages, play a key role in inflammation-mediated cartilage damage in conditions like OA. Anti-inflammatory factors can inhibit or attenuate inflammatory responses, thereby reducing the secretion of SASP and helping to alleviate chronic inflammation caused by senescent cells. Additionally, anti-inflammatory factors can regulate the survival and function of senescent cells, minimizing their harmful effects on surrounding tissues.

Inflammatory signaling pathways play a crucial role in mediating the inflammatory process in OA. Classical signaling pathways like MAPK, NF-κB, and ERK1/2 are involved, making them targets for drugs aimed at treating inflammation in OA.^96–105^ Catabolic factor stimulation activates these pathways, upregulating the expression of inflammatory genes like MMP and ADAMTS.^106^ The Wnt signaling pathway is also involved in inflammatory processes.^107^ As upstream regulators, MAPK and NF-κB pathways influence autophagy-mediated cartilage homeostasis. Therefore, regulating the autophagic process may delay OA progression.^100^ Additionally, a correlation between copper apoptosis-related genes and immune infiltration in OA patients was found through combinatorial analysis of OA transcriptome data.^108^ Given the interaction between inflammation and cellular senescence, tissues involved in the inflammatory response in OA may undergo cellular senescence.

## Cellular senescence in OA

### The concept of SASP and the relationship with disease

Age-related SASP contributes to the onset and progression of many senescence-related diseases.^109^ SASP in senescent cells affects the microenvironment via inflammatory mediators.^110^ Evidence has shown that numerous inflammatory factors in SASP may induce low-level chronic inflammation in aging tissues and accelerate organ degeneration.

Some scholars have reported that transplanting senescent cells into mice leads to age-related pathological changes and persistent physiological impairment, particularly in secretory function.^111^ Zeng et al. reported that aging-related kidney injury and inflammation regulate the RIG-I/NF-κB signaling pathway by promoting Klotho downregulation, accelerating aging in mice.^112^ Bailey-Downs et al. revealed a novel paracrine pathway leading to vascular redox imbalance, suggesting that senescence exacerbates oxidative stress and secondary low-level chronic inflammation in vivo.^113^ Additionally, several studies have found that clearing senescent cells in vivo provides varying benefits across different disease models.^114^

Numerous molecular mechanisms regulate SASP. NF-κB enhancer and C/EBP-β transcription factors play pivotal roles in regulating SASP at the mRNA level.^115^ The transcription factor GATA4 regulates SASP, and its activation depends on DNA damage regulators ATM and ATR, which activate NF-κB to promote SASP and aging. IL-1α has been shown to promote NF-κB signaling and upregulate many SASP genes.^115^ Many ROS-related factors, such as the ROS protein kinase CD1 axis, are crucial for the induction of IL-8 and IL-6, and thus the regulation of SASP.^116^ Additionally, MTOR regulates MAP kinase-activated protein kinase 2 (MAPK/APK2) and IL-1α, making it an important regulator of SASP. MAPK/APK2 can be phosphorylated by p38 to inactivate ZFP36L1, contributing to the degradation of pro-inflammatory SASP factors.^117^

**Fig. 4:**
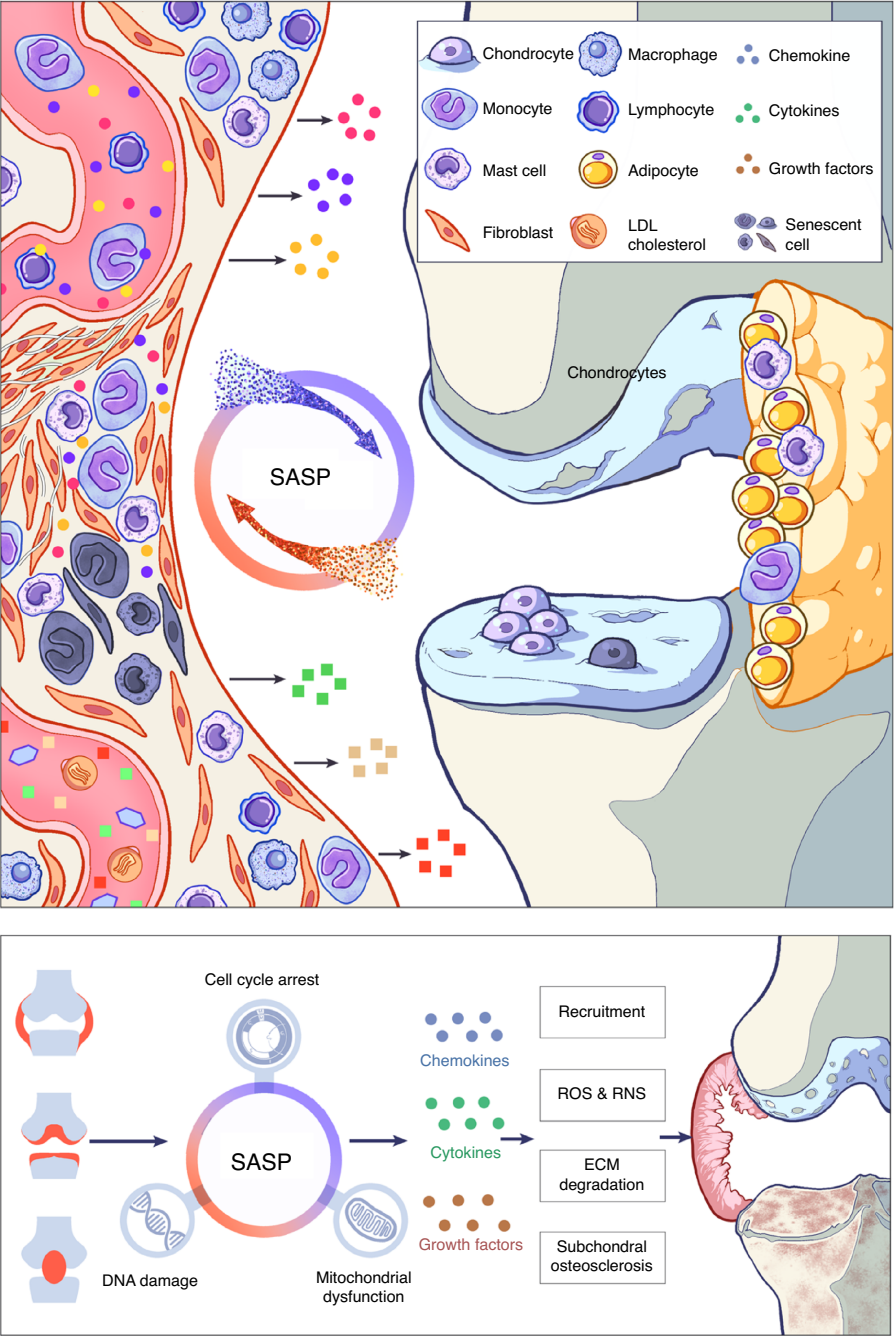
Inflammation leads to OA through SASP. Inflammation of internal joint tissues induce the hallmarks of senescence in resident cells, which further facilitates SASP (secretion of multiple bioactive factors such as chemokines, and cytokines and growth factors) and the secondary pathophysiological changes (recruitment of inflammatory cells such as macrophages, ROS&RNS, ECM degradation, and subchondral osteosclerosis) to lead to pathologies commonly found in OA (synovitis or hyperplasia, cartilage destruction, and subchondral bone sclerosis). Senescence-mediated SASP triggers or accelerates the process of inflammation-induced arthritis, exerting a cascading amplification effect

Increasing evidence supports that low-grade systemic and local inflammation play a key role in the pathogenesis of OA.^118^ Numerous studies have shown that senescence is related to the etiopathogenesis of many age-related diseases, including OA.^119^ Senescent chondrocytes have been identified in the cartilage of replacement joints. Notably, senescent cells are not restricted to chondrocytes. They are also found in other joint components, including subchondral bone, synovium, stem cells, and the infrapatellar fat pad. Researchers have developed a method to alleviate OA by preventing the aging of chondrocytes and other joint cells.^120^ This section explores the age-related phenotype of resident joint cells and examines its relationship with OA pathogenesis (Fig. 4).

### Chondrocyte senescence

Chondrocytes are the key resident cells in articular cartilage, crucial for maintaining cartilage function and contributing to OA development. Although chondrocytes have poor self-renewal ability, they retain some proliferative potential during the early stages of tissue repair.^121^ In vitro, chondrocytes promote cell division and exhibit the ability to form cell clusters.^122^ While chondrocyte senescence is correlated with OA, the mechanism linking aging and OA remains unclear.^123^

Several studies suggest that chondrocyte senescence can be triggered by factors such as nutritional deficiency, hypoxia, ROS, DNA damage, protein aggregation, damaged organelles, or intracellular pathogens. Furthermore, chondrocyte senescence and SASP trigger pathological biochemical changes in joint cells, ultimately leading to the onset and progression of OA.^124^ However, the molecular mechanisms driving chondrocyte senescence and renewal remain unclear. Evidence suggests that chondrocyte senescence can be caused by several factors. Coryell et al. reported that articular chondrocyte senescence is primarily driven by telomere erosion, cyclin-dependent kinase (CDK), and increased senescence-associated heterochromatin.^120^ Martin et al. studied the link between telomere erosion and senescence in chondrocytes, demonstrating their causal relationship.^125^ Based on the literature, we conclude that chondrocyte senescence is primarily caused by the following factors: (1) Telomere erosion. Senescent joint cells commonly exhibit telomere erosion, which strongly correlates with articular cartilage degradation. This process is driven by replication-related aging, reduced mitotic activity, and shorter telomere length.^126^ (2) Decline in mitotic activity. Senescent chondrocytes increase ROS levels via mitochondrial dysfunction and elevated senescence-related heterochromatin, promoting oxidative stress. (3) H-thymidine incorporation assay is a primary method for measuring mitotic activity.^126^ (4) Cell cycle arrest. Cellular senescence is characterized by a hyporeplicative state termed cell cycle arrest, primarily mediated by the upregulation of p53/p21/p16 pathways. Childs et al. demonstrated that replication arrest is typically triggered by DNA damage or stress signals and executed by activation of the p16 or p53 pathway.^7^ Loeser et al. reported that aging chondrocytes in OA exhibit intrinsic replicative senescence, known as cell-cycle arrest, primarily dependent on increased expression of p53, p16, p21, and other effectors.^126^ As a key participant in the DNA damage response (DDR) pathway, the p53 tumor suppressor protein is a crucial regulator of the cell cycle. The accumulation of phosphorylated p53 promotes cyclin-dependent kinase inhibitor (CDKI) activation, eventually leading to cell cycle arrest.^127^ p21, a recognized marker of senescence, is the downstream CDKI of phosphorylated p53. When p21 binds to CDK2, it inhibits the cell cycle by blocking the transition from G1 to S phase.^128,129^ Notably, p16 is highly expressed in aged chondrocytes. It induces senescence by binding CDK4 and CDK6, blocking the retinoblastoma protein (Rb), a cell cycle repressor. It mediates responses to cellular stress, such as DNA damage from radiation, telomere shortening, ROS, or oncogenic stress.^130^ Collectively, these cell-cycle inhibitors trigger apoptosis and senescence, leading to damage and loss of articular cartilage, contributing to OA development.

Numerous studies explore the mechanisms underlying chondrocyte senescence. In addition to the molecules mentioned above, including p53, p16, and p21, which are involved in cell cycle arrest, other key factors contribute to chondrocyte senescence and OA progression. Li et al. found that Sirt6 reduces chondrocyte aging and OA progression by interacting with STAT5, inactivating the IL-15/JAK3/STAT5 pathway.^131^ Varela-Eirín et al. demonstrated that Cx43 promotes chondrocyte-mesenchymal transition and reduces cellular senescence by increasing Twist-1 nuclear translocation during OA progression.^132^ Horváth et al. reported that chondrocyte senescence in OA is induced by Sox-9, aggrecan, and Col2a1 suppression, while increased expression of HIF-2α, RunX2, and MMP-13 inhibits the transition to senescence.^133^ However, the role of senescence in compromising joint stability and function in OA remains unclear, and further investigation is urgently needed.

The extracellular matrix (ECM) provides structural support, creates a favorable environment for various cell types, and influences key cellular processes.^134^ Changes in senescence-related ECM proteins profoundly affect homeostasis and physiology. Studies show that metabolic disorders and increased ECM catabolism in articular cartilage are key factors in OA development.^135^ Guo et al. studied the relationship between mitochondrial DNA dysfunction and OA, finding that STING activates the NF-κB signaling cascade to promote senescence, inducing secondary ECM degradation in OA.^136^ Lu et al. demonstrated that fibroblast growth factor 21 (FGF21) alleviates chondrocyte senescence and ECM impairment in OA via the SIRT1-mTOR signaling pathway.^137^ FGF21 administration has been shown to alleviate both chondrocyte senescence and ECM catabolism. While the molecular mechanisms driving ECM degradation and OA remain unclear, increased expression of inflammatory mediators from cartilage aging and SASP may be key factors in OA development. Notably, a balance exists between chondrocyte senescence and the metabolic regulation of inflammation, as demonstrated by recent findings. In a seminal study, Arra et al. found that chondrocytes undergo metabolic shifts in inflammatory states involving NF-κB activation, which reprograms cellular glycolysis and lactate dehydrogenase A.^97^ Lactate dehydrogenase A promotes ROS-induced catabolism. Inflammation and senescence converge on IκB-ζ, a key mediator downstream of NF-κB, regulating RANKL, inflammation, catabolism, and SASP gene expression to program chondrocytes into an “inflammatory phenotype”.^123^ This indicates a close link between metabolic regulation of cellular senescence and inflammation, suggesting that cellular senescence can be alleviated by correcting metabolic imbalances.^138,139^

Notably, SASP occurs not only in chondrocytes but also in bone and synovial cells, possibly induced by chondrocyte-centered intercellular communication.^140,141^ Cellular aging is characterized by DNA damage, mitochondrial dysfunction, and permanent cell cycle arrest, ultimately leading to SASP. SASP leads to the release of pro-inflammatory molecules into neighboring tissues and cells. Studies suggest that chondrocytes stimulate osteocytes and synovial fibroblasts, affecting their limited regenerative potential.^20^ Thus, we believe that senescent chondrocytes promote OA through intercellular communication, including chronic low-grade inflammation known as “inflammsenescence”. Some studies have explored the mechanisms by which chondrocytes alter their surrounding environment and cells. These findings suggest that the spread of senescence relies heavily on SASP development. Several studies have shown that senescent chondrocytes spread senescence to surrounding tissue through SASP, involving the secretion of cytokines, growth factors, chemokines, and other bioactive factors to influence signaling in an autocrine or paracrine manner. Jeon et al. documented that elevated activation of the IL-6–STAT3 signaling pathway in the synovium of OA patients induces fibroblast aging, indicating bystander effects that lead to secondary aging and SASP in neighboring cells.^142^ Coppé et al. suggested that SASP manipulates the surrounding microenvironment through paracrine signaling pathways.^110^ Zhu et al. reported that senescent chondrocytes produce and secrete bioactive molecules, including chemokines, cytokines, matrix-degrading enzymes (MMPs), and growth factors, facilitating cell-cell contact through gap junctions and further inducing neighboring cell aging.^143^ Collectively, these findings suggest that senescent chondrocytes activate SASP to secrete bioactive factors via paracrine pathways, transforming neighboring microenvironments and exerting systemic effects on the entire joint.

Additionally, evidence suggests that chondrocytes promote intercellular communication by releasing extracellular vesicles (EVs) into the synovial microenvironment, triggering senescence in bystander cells.^142,144^ Jeon et al. investigated the differences between EVs and SASP.^142^ They evaluated EVs production from senescent chondrocytes in arthritic cartilage and found a positive correlation between EVs production and the number of senescent cells. EVs are crucial cellular messengers that transfer senescence signals from senescent cells, playing a key role in senescence propagation and age-related OA. Mechanistically, EVs transfer senescence markers to bystander cells and inhibit cartilage regeneration by altering the expression of miR-34a, −92a, −24, −186, and −150. In summary, EVs produced or secreted from synovial fluid and senescent chondrocytes may serve as key mediators of senescence progression and OA pathology.

Senescence propagation occurs in other tissues. Liu et al. showed that bone-marrow adipocytes (BMAds) spread senescence to surrounding bone and bone marrow tissue through SASP, increasing oxylipin synthesis and expression of key senescence genes.^145^ They also demonstrated that oxylipin and its downstream effector PPARγ induce the expression of senescence-related genes, which in turn promote oxylipin synthesis in BMAds, forming a positive feedback loop. Additionally, Nelson et al. reported that senescent MRC5 fibroblasts induced secondary senescence in bystander fibroblasts through the production and secretion of bioactive factors, including cytokines, MMPs, growth factors, and ROS, via gap junction-mediated cell-cell contact.^146^ They further demonstrated that continuous exposure to senescent MRC5 fibroblasts induced senescence in neighboring fibroblasts, and that senescent hepatocytes aggregate in vivo. Waters et al. reported that senescent lung fibroblasts (LFs) induced a senescent-like phenotype in non-senescent LFs when exposed to alveolar epithelial cells (AECs) in vitro.^147^ This study offers a possible explanation for the abnormal abundance of senescent cells in the lungs of patients with idiopathic pulmonary fibrosis. We propose that senescent cells, including chondrocytes and other bone-resident cells, may stimulate secondary senescence and damage the local environment through SASP, senescence-associated EVs, and gap junction-mediated cell-cell contact. These hypotheses require further experimental validation and theoretical support.

### Stem cell senescence

In recent years, mesenchymal stem cell (MSC)-based therapy has emerged as a complementary approach to treat OA. MSCs offer advantages such as easy accessibility, simple isolation, favorable proliferation, and multilineage differentiation potential, making them an excellent resource for OA treatment. Rizzo et al. demonstrated that MSCs or MSC-derived EVs combined with senolytic agents regulate intercellular communication, providing targeted therapeutic effects against senescent cells and SASP in OA.^148^ Based on tissue specificity, MSCs in the joint include synovial MSCs, adipose-derived MSCs, and BMSCs. Viable MSC-based therapies are in preclinical models and clinical treatments for OA, including local injection of MSCs, MSC-derived EVs, and MSC-loaded scaffold implants.

Understanding the mechanisms underlying MSC application in OA treatment is crucial. The immunomodulatory effect of MSCs is key in treating OA. Inflammatory factors released by senescent cells can activate MSCs, which then secrete PGE2, IDO, and NO to inhibit inflammatory cells and alleviate OA.^149,150^ MSC-derived cytokines regulate the synthesis and breakdown of metabolic factors, inducing anti-inflammatory factor expression in the synovium.^151^ Additionally, MSC-derived cytokines promote chondrocyte proliferation and ECM synthesis, repairing damaged bone and cartilage.^152^

MSC senescence also influences the development and progression of OA.^153–155^ Ye et al. suggested that MSC senescence is closely related to organic aging and the occurrence of aging-associated diseases, including OA.^156^ Čamernik et al.^157^ demonstrated that MSC depletion and functional decline in subchondral bone may contribute to OA development. Cao et al.^158^ showed that aging chondrocytes reduce MSCs’ natural potential to differentiate and proliferate, driving apoptosis of senescent chondrocytes and promoting OA. MSC senescence leads to significant changes in cell phenotype, including telomere shortening, altered cell surface markers, epigenetic changes, flattened or enlarged cell morphology, impaired differentiation potential, and decreased proliferation capacity.^159^ Nevertheless, deeper understanding of stem cell senescence mechanisms in OA is needed, and exploration of anti-aging agents to treat OA is essential.

### Synovium senescence

Pathological changes in synovial of OA, such as proliferative and fibrous synovitis, are key manifestations of the disease. In contrast, several studies have demonstrated that SASP factors in synovial fibroblasts trigger OA-related changes, including joint inflammation, cartilage degeneration, subchondral osteosclerosis, and ECM degradation.^120,160,161^ Coppé et al. reported that nutrient deficiency, hypoxia, DNA damage, reactive oxygen species (ROS), damaged organelles, or intracellular pathogens can activate various cytokines, such as IL-1, IL-6, and IL-17.^101^ These cytokines, typical SASP factors, can promote synovial fibroblast senescence and contribute to joint degeneration. Therefore, a deeper understanding of the relationship between SASP in synovial fluid and OA pathogenesis could elucidate the role of SASP factors in joint tissue degeneration.

The SASP of intra-articular cells plays a crucial role in the degeneration of the surrounding ECM. Xu et al. performed senescent cell transplantation and discovered that introducing aging cells into the knee joint causes leg pain, impaired mobility, and radiological as well as histological alterations characteristic of OA.^144^ Del Rey et al. reported that increased inflammation in rheumatoid arthritis tissue leads to the premature accumulation of senescent synovial fibroblasts.^141^ Senescent cells in the synovium can induce fibrous synovitis, ECM degeneration, and cartilage damage, indicating that the SASP of synovial cells can significantly alter the joint’s microenvironment.^162^ The degradation of the surrounding ECM is primarily mediated by: (1) SASP-released cytokines that induce the breakdown of ECM proteins, including collagen, sulfated proteoglycans, and fibronectin, by regulating the expression of IL-1, IL-6, and IL-17 in cartilage.^161^ (2) The action of MMPs and ADAMTS (a disintegrin and metalloproteinase with thrombospondin motifs), such as MMP13 and ADAMTS-5. The depletion of ADAMTS plays a key role in ECM degradation and is associated with chondrocyte senescence and OA progression.^163^ (3) Senescent chondrocytes enhance the secretion of EVs, promoting intercellular communication in bystander fibroblasts and inducing a bystander effect that drives the senescence of neighboring tissues.

### Infrapatellar fat pad senescence

The patellar fat pad, the largest soft tissue structure in the knee joint, is situated between the femoral condyle, tibial plateau, and patella. Its flexible and displaceable structure helps fill the anterior gap of the knee joint, absorbing force, reducing overload, and protecting the joint.^164^ Additionally, the fat pad promotes uniform distribution of synovial fluid, limits excessive knee movement, and provides lubrication. Fat pads are also a source of stem cells, inflammatory factors, and neuropeptides. Inflammation of fat pads has been linked to cartilage loss and ECM degradation, indicating their potential role in driving the development and progression of OA.^165,166^

Studies have demonstrated that aging adipose tissue is strongly associated with several diseases, including cardiovascular and metabolic diseases.^167^ OA is now considered a disease of the entire “joint organ,” and substantial evidence suggests that the patellar fat pad plays a role in knee OA development.^168^ The infrapatellar fat pad is associated with cartilage lesions and elevated inflammatory factor production, contributing to the development of knee OA.^169^ Favero et al. compared the aging infrapatellar fat pad in OA patients to that in non-OA patients, confirming its crucial role in OA pathology due to its susceptibility to inflammation, vascularization, and fibrosis.^165^

The mechanisms by which the infrapatellar fat pad contributes to this process have been partially elucidated. This mechanism appears to be multifactorial, potentially involving a pro-inflammatory state related to aging, commonly referred to as “inflammaging”.^29^ Aging-related inflammation can occur both systemically and locally. Studies indicate that chronic low-grade inflammation in adipose tissue is a key mechanism driving the progression of OA.^167^ Researchers have investigated obesity-related changes in systemic and adipose tissue-resident immune cells, discovering that metabolic disorders in aging adipose tissue ultimately lead to an inflammatory phenotype and tissue remodeling.^167,170^ Aging in the patellar fat pad can trigger low-grade inflammation, disrupting the balance between acute and chronic inflammation, ultimately contributing to joint damage.^171^ Additionally, adipocyte hypertrophy and dysfunction in aging adipose tissue are associated with shorter telomere length, altered cell proliferation, and accelerated OA progression.^11^ Macroscopically, the patellar fat pad interacts with surrounding tissues, including cartilage, subchondral bone, and synovium, playing a significant role in OA pathology.^165,166^ Further research is required to gain a deeper understanding of this process and its underlying mechanisms. Moreover, effective strategies must be developed to prevent these degenerative processes.

### Immunosenescence

Immunosenescence refers to the dysfunction of both the innate and adaptive immune systems during aging, characterized by reduced T and B cell production and the accumulation of atypical cell subsets.^172^ The immune system is typically activated by PRRs, which initiate inflammatory responses to infections.^173^ Under normal conditions, innate and adaptive immunity are tightly regulated, with damage promptly followed by repair. However, as joint cells age, various immune dysfunctions emerge within the body. This process involves both innate and adaptive immune responses, with key features including thymic degeneration and reduced T cell production; shifts in T cell populations, such as increased memory T cells and decreased naïve T cells; impaired immune surveillance; poor vaccine response and increased infection susceptibility; higher incidence of autoimmune diseases and cancer; and senescence-associated dysregulated secretion of pro-inflammatory cytokines, chemokines, and proteases. Additionally, changes in metabolic and epigenetic pathways contribute to immune system and T cell aging.^172,174,175^ During bone and joint aging, chronic immune responses can cause cartilage loss, ECM degradation, and subchondral sclerosis.^176^ This section of the review aims to outline the key effector cells and molecules while exploring the potential mechanisms of OA-related immunosenescence.

#### Immune cell senescence

Senescence-associated deterioration of innate and adaptive immunity in joints impairs immune defenses, leading to persistent low-grade chronic inflammation. This promotes the accumulation of senescent phenotypes and the elevated production of pro-inflammatory factors, leading to SASP in resident tissues (cartilage, synovium, subchondral bone) and increasing susceptibility to OA.^177,178^ A variety of cells, including macrophages, fibroblasts, and mast cells, are involved in the development of immunosenescence-related OA. Evidence indicates that monocytes/macrophages play a crucial role in OA-related inflammation and can be activated to produce excessive cytokines, MMPs, and growth factors, contributing to OA pathology.^179^ Senescent immune cells, such as CD28-T cells and CD14^+^CD16^+^ monocytes, are more abundant in OA patients than in healthy controls, contributing to severe chronic inflammation in OA.^120,172^ Studies demonstrate that depleting macrophages in cocultures with synovial cells from OA patients leads to a significant reduction in cytokines (e.g., IL-1, IL-6, TNF-α) and MMPs (MMP1, MMP3, MMP9, MMP13), modulating inflammation and OA progression.^180^ This process forms a closed loop, where inflammation drives immune cell senescence, and senescent immune cells, in turn, perpetuate chronic inflammation.

#### Chondrocyte-related immunosenescence

Chondrosenescence refers to the senescence-driven dysfunction of chondrocytes, which impairs cartilage function in OA. Although chondrocytes are not part of the immune system, they can express various innate immunity receptors and produce inflammatory effectors during OA progression. When aging chondrocytes are activated in OA, they upregulate pro-inflammatory factors such as TNF-α, IL-1, and IL-6 through the complement system.^181^ Additionally, senescent chondrocytes upregulate C5a receptor expression in response to the inflammatory microenvironment, further worsening articular cartilage degeneration, subchondral osteosclerosis, and synovial hyperplasia.^178,182^ While current studies primarily emphasize the pro-inflammatory effects of senescent chondrocytes, other joint-resident cells, including synovial cells, fibroblasts, and subchondral bone cells, also play a significant role in OA pathology.

#### Cytokine senescence

Immunosenescence is more pronounced in patients with OA compared to healthy controls, marked by increased pro-inflammatory cytokine production. Cytokine senescence refers to abnormal cytokine levels and activity, indicating the transformation of normal cells into a senescent, imbalanced state. Previous studies have highlighted the significant roles of cytokines, particularly TNF-α, IL-1, IL-6, and IL-17, in the initiation and progression of OA.^183^ A growing number of studies have shown elevated IL-17 and IL-18 expression in OA synovial fluid, identifying them as key cytokines in OA pathology.^184,185^ These studies suggest that cytokine senescence can trigger inflammatory reactions and tissue degeneration, driving the onset and progression of OA. Additionally, inflammation in the synovium, cartilage, and patellar fat pad leads to the secretion of inflammatory factors that infiltrate the articular cartilage, increasing the release of metabolic mediators.^186^ Evidence indicates that cytokines play crucial roles in promoting premature senescence in surrounding young cells. Nakajima et al. reported that IL-6 regulates senescence in multiple systems by forming IL-6/sIL-6Rα complexes with STAT3, inducing premature senescence in human fibroblasts.^187^ However, the underlying mechanisms remain unclear, and the role of cytokine senescence in OA is still debated, necessitating further research.

#### Complement system senescence

The complement system plays a vital role in the body’s defense mechanisms and is critical in protecting against diseases. An increasing number of studies show that complement activation products are elevated in the serum and synovial fluid of OA patients.^188^ Specifically, the complement system in aging synovial cells can be activated via the classical, alternative, and lectin pathways, forming a membrane attack complex (MAC) that induces synovial fluid inflammation in OA.^189^ Wang et al. conducted proteomic and transcriptomic analyses of synovial fluid and synovium in OA patients, finding that complement activation and MAC-mediated pathways play crucial roles in OA pathology.^190^

#### Chemokine senescence

In addition to cytokines, chemokines are also involved in the pathogenesis of OA. Numerous studies have shown that the levels of CC motif ligands 2 (CCL2), CCL3, CCL4, and CCL5 are elevated in the serum and synovial fluid of OA patients compared to those without OA and are positively correlated with disease severity.^191^ Tsuchida et al. reported that age-related stress contributed to OA development, partly due to the senescence of chemokines such as CCL2, CCL4, and GROα.^192^ These chemokines induced macrophage recruitment, inflammation, and pain. Zhao et al. demonstrated that multiple chemokines are involved in the inflammatory and catabolic processes of chondrocytes, potentially recruiting inflammatory cells such as neutrophils and monocytes to accelerate OA pathology.^193^ Acosta et al. demonstrated that the chemokine receptor CXCR2 (IL8RB) promotes senescence by binding to CXCR2 in a p53-dependent manner.^194^ In conclusion, chemokine-related senescence serves as an important mediator and functional pathway in joint senescence and OA pathology, either independently or in conjunction with other biological factors. This warrants further attention in understanding aging-induced OA.

## New strategy for treating oa from the cellular senescence perspective

Various factors influence cellular senescence, and the development of anti-senescence drugs or strategies targeting the physiological mechanisms of senescence offers new therapeutic approaches for delaying senescence-related chronic diseases. Rapamycin, a key anti-senescence drug, induces autophagy to counteract cellular senescence caused by ROS upregulation due to increased inflammatory cytokines. Its mechanism involves promoting antioxidant protein expression by enhancing Nrf2/Keap1 signaling.^195^ A recent study demonstrated that coumarins, which induce mitochondrial autophagy, improve mitochondrial function and extend lifespan by inhibiting the activation of the nuclear hormone receptor DAF-12/FXR.^196^ L-glutamine, a common amino acid in human blood, inhibits NF-κB activity.^197^ This inhibition occurs via the upregulation of long non-coding RNA NKILA expression, regulated by the TGF-β1/SMAD2/3 pathway, leading to reduced expression of NO synthase, COX-2, and MMP-13. Oleanolic acid rescues mitochondrial ultrastructural abnormalities, scavenges free radicals, and regulates P450COX, thereby modulating mitochondrial integrity and autophagy in senescent cells. This modulation effectively prevents cardiac senescence by upregulating FUNDC-dependent mitochondrial autophagy, mediated by the E3 ligase MARCH5.^198^ Similarly, metformin, a widely known hypoglycemic agent, increases autophagy in T-cells and improves mitochondrial bioenergetics, restoring senescence-related inflammation to a normal state.^199^ Mitochondrial dysfunction in senescent cells increases harmful substances like ROS. Additionally, resveratrol and EVs from adipose stem cells counteract the adverse effects of ROS production.^200,201^ Resveratrol specifically reduces ROS levels, attenuates IL-1-induced SASP, and delays OA progression via the ROS/NF-κB axis in the ACLT rat model. Stem cell-derived EVs regulate senescence-related signaling pathways through functionally important miRNAs. As a hallmark of cellular senescence, SA-β-gal serves as a drug initiator, selectively releasing gemcitabine after activation of the prodrug SSK1, which removes senescent cells.^202^ Upon cellular senescence, cell-free mitochondrial DNA accumulates, enhancing immunogenicity. Additionally, the activated type I interferon response is crucial for maintaining SASP. Thus, anti-senescence can be achieved by targeting senescence-associated inflammation, using agents like the nucleoside reverse transcriptase inhibitor lamivudine, senolytics, or melatonin.^46,203,204^ Beyond conventional drugs, DNASE2A may enhance the clearance of excess extra-nuclear DNA in senescent cells by triggering autophagy, reducing innate immune response and SA-β-gal activity.^41^ Notably, these anti-senescence drugs may involve multiple mechanisms, many of which remain poorly understood, partly due to research limitations and potentially undiscovered pathways.

**Fig. 5:**
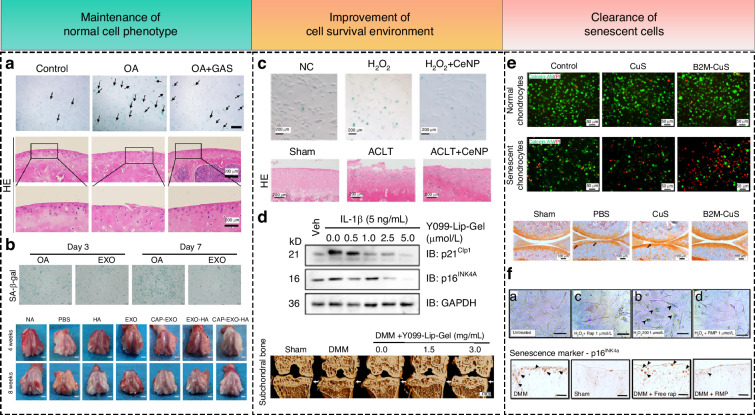
The therapeutic efficacy of representative anti-senescence drugs with different mechanisms of action in vitro and in vivo. **a** Gastrodin regulates phosphorylation of the PI3K-AKT pathway via SIRT3, reduces SA-β-gal positive staining in IL-1β-treated chondrocytes, and reverses cartilage destruction in the OA rat model.^226^ Permission of reuse obatained from copyright holder Elsevier. **b** Chondrocyte cultures supplemented with exosomes from umbilical cord MSCs significantly reduce SA-β-gal positive staining of OA chondrocytes and improve articular cartilage bulk structure.^213^ Permission of reuse obtained from copy right holder American Chemical Society. **c** Cerium dioxide nanoparticles reduce the percentage of SA-β-gal-positive cells in H_2_O_2_-treated synoviocytes and protect articular cartilage by scavenging ROS and inactivating the NF-κB signaling pathway.^207^ Permission of reuse obtained from copyright holder MDPI. **d** Multi-kinase inhibitor YKL-05-099 inhibit MAPK and NF-κB signaling activation by affecting kinome phosphorylation, reduce IL-1β-induced chondrocyte senescence, decrease the level of senescence markers p21^Clp1^ and p16^INK4A^ in chondrocytes, and prevent subchondral bone loss effectively.^212^ Permission of reuse obtained from copyright holder The Author(s). **e** Small copper sulfide nanoparticles functionalized with anti-beta-2-microglobulin antibodies specifically induce apoptosis in senescent chondrocytes and prevent articular cartilage damage.^233^ Permission of reuse obtained from copyright holder The Author(s). **f** Rapamycin decreases the levels of senescence markers in H_2_O_2_-stimulated human chondrocytes and reduces joint P16^INK4a^ positivity in the mouse OA model by upregulating autophagy.^210^ Permission of reuse obtained from copyright holder The Author(s)

These anti-senescence drugs and strategies have shown efficacy, marking a breakthrough in the treatment of cartilage-damaging diseases like OA. While exercise and dietary strategies were discussed previously, this section focuses on recent advancements in anti-senescence pharmacological treatments for OA. First, several types of senescent cells and tissues within the joint cavity, including chondrocytes, stem cells, and synovial cells, can be targeted for drug intervention. However, drug specificity depends more on the cellular state than on tissue specificity, and cell state-specific drugs target senescent cell markers.^205^ Second, based on their effects, these drugs can be categorized into three types: maintaining normal cell phenotypes, improving the cell survival environment, and clearing senescent cells (Fig. 5). Specifically, maintaining normal cellular phenotypes involves enabling senescent cells to continue their biological functions; improving the cellular environment targets senescence-associated inflammation; and drugs for senescent cell elimination focus on enhancing autophagy and inducing apoptosis. Majority of studies focus on senescent chondrocytes in OA, but it is important to recognize that multiple cell types contribute to OA pathology. Therefore, it is crucial to investigate cellular senescence in other cell types, including adipose mesenchymal stem cells and synoviocytes.^206,207^ Recent studies on anti-senescence drugs for OA treatment are summarized in Table 2. Additionally, some techniques can also achieve anti-senescence effects. Pretreatment of MSCs from elderly OA patients with chondrogenic differentiation medium followed by normal growth medium rejuvenated senescent MSCs and significantly improved rabbit OA pathology. Moreover, therapeutic efficacy correlated with cell number.^208^

**Table 2:** Anti-senescence drugs for OA

Drug	Mechanism	Validation in vitro	Validation in vivo	Limitation	Ref.
Maintenance of normal cell phenotype					
β-Hydroxybutyrate	Upregulated PTEN expression and inhibited the downstream P13K/Akt signaling pathway	Improved H_2_O_2_-induced senescent phenotype and proliferative activity of chondrocytes derived from OA patients	Improved COL2A1 expression and maintained cartilage morphology in OA rats Inhibited MMP13, P16 and P21 expression	–	^225^
Gastrodin	Upregulated Sirtuin3(SIRT3) expression and downregulated protein phosphorylation of the PI3K-AKT pathway	Attenuated IL1-β-induced chondrocyte senescence, mitochondrial homeostasis imbalance	Ameliorated cartilage erosion, chondrocyte senescence and OA injury in rat knee joints	–	^226^
Vildagliptin	upregulated SIRT1 expression and attenuated AMPK-SIRT-p53 acetylation	Attenuated chondrocyte senescence and senescence-associated protein expression induced by TNF-α and ameliorated chondrocyte cell cycle arrest in G1 phase	–	(1) Validation of in vivo models was absent (2) Single stimulus source cannot fully model OA pathogenesis	^227^
Parathyroid hormone-related protein-derived peptide C-terminal fraction	Reduced activation of NF-κB	Reduced senescence marker expression levels, number of γH2AX foci, and inflammatory response in IL-1β-induced OA osteoblasts, and enhanced osteoblast mineralization	–	Lack of comparison of OA and healthy osteoblasts from same-age donors	^228^
Exosomes from umbilical cord mesenchymal stem cell sources	Involved in regulating the p53 signaling pathway	Inhibited the expression of OA chondrocyte senescence genes, restored the viability of senescent OA chondrocytes, and promoted the synthesis of cartilage matrix	"Two-Phase" release system enhanced exosome therapeutic efficiency and retention time	(1) Exosomes have a complex composition and their therapeutic role still needs to be explored (2) Intelligent release needs to be matched to disease	^213^
Improvement of cell survival environment					
Butorphanol tartrate	Inactivated NF-κB and STAT3	Reduced percentage of SA-β-gal positivity and G0/G1 phase in TNF-α-induced human articular chondrocytes, reduced p21 protein levels, elevated telomerase activity, and neutralized TNF-α-induced inflammatory response	–	Effects of drugs in vivo not explored	^229^
Heme oxygenase-1	Reduced production of relevant inflammatory and catabolic mediators involved in OA pathophysiology	Upregulated osteogenic differentiation and mineralization gene expression, downregulated MMP and senescence-related gene expression, and inhibited NF-κB activation	–	Validation of in vivo models was absent	^230^
Ceria Nanoparticles	scavenged ROS and inactivated the NF-κB signaling pathway	Removed synoviocyte senescence and inhibited SASP triggered by H_2_O_2_, attenuated senescence and inhibited SASP in multiple passaged synoviocytes, and inhibited NF-κB pathway activation in senescent synoviocytes	Reduced ROS content attenuates synovial cell senescence and SASP expression in a rat OA model constructed by ACLT surgery	(1) Failed to measure intra-articular SASP protein concentrations (2) Failure to study effects on other tissues such as chondrocytes	^207^
multi-kinase inhibitor YKL-05-099	Inhibited MAPK and NF-κB signaling activation	Suppressed IL-1β-induced inflammation and catabolism, promoted chondrocyte anabolism, and inhibited senescence inducer and SASP factor expression	Attenuated histological damage to cartilage in mice models of OA, inhibited subchondral bone loss and osteoclast formation	(1) Lack of cartilage-targeted type (2) Failed to study the mechanical properties of hydrogels (3) Failed to evaluate hydrogel drug delivery systems system in vivo safety systematically	^212^
Clearance of senescent cells					
senolytic	Induced apoptosis selectively in some senescent chondrogenic progenitor cells	Increased proliferation of senescent chondrogenic progenitor cells, accelerated cartilage regeneration from chondrogenic progenitor cells, and significantly reduced supernatant IL-1β levels	Significant restore of articular cartilage integrity and corrected abnormal subchondral bone sclerosis in combination with arthrodesis	(1) Failed to use synovial fluid to assess levels of inflammatory factors (2) Failed to explore other mechanisms of action of the drug	^231^
Navitoclax (ABT263)	Induced apoptosis in senescent cells	Removed senescent rat chondrocytes induced by ionizing radiation in a dose-dependent manner, eliminated SA-β-gal-positive senescent cells in chondrocytes and cellular microcolonies, and promoted the chondrogenic phenotype	Attenuated cartilage and subchondral bone damage in a rat model of post-traumatic OA	(1) Failed to study potential effects on other tissues such as synovium (2) The period of in vivo experiments was short (3) Failed to elucidate the apoptosis signaling pathway	^205^
Navitoclax (ABT263)	Induced apoptosis in senescent OA synovial MSCs cells	Significantly reduced SA-β-gal positivity in synovial MSCs derived from OA patients and expression of B-cell lymphoma 2	–	(1) Failed to validate in vivo therapeutic efficacy (2) Absence of quantification of released SASP factors	^232^
anti-beta-2-microglobulin antibodies	Induced apoptosis in senescent chondrocytes by peroxidase-like activity	Targeting senescent chondrocytes for elimination and upregulated cartilage-related gene expression	Removed senescent cells in the joints of OA mice constructed by DMM surgery and promoted cartilage regeneration	–	^233^
Rapamycin	Upregulated autophagy	Induced autophagy in primary human articular chondrocytes, reduced the percentage of senescent cells induced by H_2_O_2_, and maintained the production of sulfated glycosaminoglycans in pressurized microcosm cultures	Effectively attenuated cartilage damage and inflammation in a post-traumatic model of OA in mice	–	^210^
Rapamycin	Activated autophagy	Induced chondrocyte autophagy in a dose-dependent manner, prevented chondrocyte senescence under two stress conditions, and maintained sulfated glycosaminoglycan production in 3D cultures	Microcarrier platform increased drug residence time in the joints	Failed to test effects in preclinical models of OA	^211^
Fibroblast growth factor 21	Upregulated chondrocyte autophagic fluxes	attenuated apoptosis, senescence and extracellular matrix catabolism in chondrocytes, which involved activation of the SIRT1-mTOR signaling pathway	Inhibited pathology in DMM surgically constructed OA mouse models	(1) In vitro experiments can only partially respond to OA pathology (2) The mechanism of action on chondrocytes was unclear	^137^
Spermidine	Increased expression of acetyltransferase EP300 and enhanced cellular autophagy	Activated autophagy in human and mouse chondrocytes, increased chondrogenic markers in mouse chondrocytes and human OA chondrocytes	–	–	^234^
Strontium	Improved autophagy in fibroblast-like synoviocytes via the AMPK/mTOR/LC3B-II signaling axis	Inhibited fibroblast-like synoviocyte senescence and significantly reduced mRNA levels of SASP	Attenuated pain-related behaviors and inhibited pathological processes in DMM-constructed OA mice	Failed to explore ion concentrations and different intracellular signaling pathways	^235^
Metformin	activated the AMPK/mTOR-dependent autophagy pathway	Increased survival and reduced senescence of adipose-derived mesenchymal stem cells, reversed excessive ROS production and DNA damage induced by H2O2	Inhibited pathologic progression and reduced pain in DMM surgically constructed OA mice	Inherent shortcomings of stem cell therapy, including frequency of injections and number of cells	^206^

In vitro experiments have demonstrated promising results with the use of anti-aging drugs alone. However, it is crucial to note that these drugs exhibit dose-dependent effects, potentially leading to high toxicity and damage to non-senescent cells.^202,209^ Therefore, the potential toxicological effects of anti-aging drugs in vivo are a major concern. To address this, it is necessary to achieve high local drug concentrations and prolonged duration of action, while ensuring biosafety. Various drug delivery systems for anti-aging therapies have been developed, including oral administration and intra-articular injections. Dhanabalan et al. developed a post-traumatic OA mouse model via medial meniscus destabilization and loaded rapamycin into polylactic acid-glycolic acid (PLGA) particles for slow drug release.^210^ The study demonstrated that intra-articular injections administered every 3 weeks effectively treated early OA in mice. Previous studies have addressed the issue of systemic toxicity resulting from frequent injections. Polymer particle-based drug delivery systems were shown to maintain a joint residence time of 19 days, which is critical for clinical translation.^211^ Wan et al. developed nanoliposome-based thermosensitive hydrogels that demonstrated promising results in reducing kinase inhibitor-induced cytotoxicity and enhancing protein kinase inhibitor performance.^212^ The therapeutic efficacy and retention time of exosomes were significantly enhanced using a two-phase system consisting of a chondrocyte-targeted polymer membrane and thiolated hyaluronic acid gel.^213^ This cell-free therapeutic strategy effectively restored senescent chondrocytes.

## Conclusions and prospects

Our understanding of OA pathogenesis is continuously advancing, shifting focus from cartilage damage alone to the involvement of subchondral bone, synovium, infrapatellar fat, and other joint tissues. Recent studies have revealed a correlation between inflammation and cellular senescence, suggesting that cellular senescence plays a key role in the inflammatory response. The cross-talk between cellular senescence and inflammation offers a novel perspective on OA pathogenesis and the development of therapeutic strategies for cartilage-damaging diseases like OA. This review synthesizes insights from recent studies, shedding light on the role of cellular senescence in OA and its underlying mechanisms. While the role of cellular senescence in driving inflammatory responses is increasingly clear, further studies are needed to unravel the underlying molecular mechanisms linking senescence and inflammation. Additionally, several critical issues must be addressed before the clinical translation of these therapies can be realized:

1. Distinguishing normal, physiologically senescent, and pathologically senescent cells in joint tissues requires the development of novel techniques. This distinction is essential for understanding and targeting the harmful effects of pathological senescence.
2. Early Identification and Intervention: While much research has focused on mitigating the effects of established senescent cells, there is a growing need to identify and address abnormal senescence before pathological changes occur. Enhanced methods for precise identification and early intervention are crucial.
3. Numerous studies have used surgery or intra-articular drug injections to establish animal models of OA. However, it is crucial to recognize that these models do not fully replicate the natural pathological progression of OA associated with aging. Therefore, future research should prioritize investigating anti-cellular senescence therapies in aging models or in elderly OA patients.
4. The pharmacokinetics of most drugs aimed at rescuing or eliminating senescent cells in the joint cavity remain unclear. Additional high-quality in vivo data are required for further exploration.
